# Potential mechanism of Taohong Siwu Decoction in uterine fibroid treatment based on integrated strategy of network pharmacology and experimental verification

**DOI:** 10.1186/s13020-023-00809-6

**Published:** 2023-08-02

**Authors:** Shasha Shi, Li Luo, Fu Peng, Chenghao Yu

**Affiliations:** 1 The Institute of Integrative Medicine, Shaanxi University of Traditional Chinese Medicine, Xianyang, 712046 China; 2 State Key Laboratory of Southwestern Chinese Medicine Resources, Chengdu University of Traditional Chinese Medicine, Chengdu, 611137 China; 3 The Basic Medical College, Chengdu University of Traditional Chinese Medicine, Chengdu, 611137 China; 4 The west China School of Pharmacy, Sichuan University, Chengdu, 610041 China

**Keywords:** Taohong Siwu Decoction, Uterine fibroids, PI3K, AKT pathway, miR-21-5p, PTEN axis

## Abstract

**Background:**

Taohong Siwu Decoction (THSWD) is a widely prescribed Traditional Chinese Medicine (TCM) for treating gynecological diseases. It is used to treat uterine fibroids (UF) in China, while its potential therapeutic effects and mechanism are unknown.

**Methods:**

The present study used network pharmacology to identify PI3K/AKT as one of the main THSWD signaling pathways that can be targeted to treat UF. The potential binding sites of miR-21-5p to PTEN were predicted using online databases. We were able to establish a UF rat model successfully. We selected the 15% THSWD serum after preparing THSWD drug-containing serum to culture tumor tissue-derived cells. These studies enabled us to assess the role of THSWD in UF improvement.

**Results:**

In vivo, we observed that low, medium, and high doses of THSWD improved histological changes in UF rats by increasing the expression levels of PTEN and miR-21-5p in their uterus while decreasing the expression levels of p-PI3K, p-AKT, and miR-21-5p. Treatment with THSWD medicated serum (15%) effectively inhibited the proliferation of cells derived from human UF and promoted apoptosis in vitro. PI3K phosphorylation, Akt phosphorylation, and miR-21-5p expression were decreased, while PTEN and cleaved caspase-3 were increased. These findings were reversed by administering 740 Y-P (a PI3K/Akt pathway agonist) and a miR-21-5p mimic. In addition, the double luciferase reporter gene assay confirmed the targeted binding relationship between miR-21-5p and PTEN.

**Conclusions:**

THSWD inhibited the expression and activation of the PI3K/AKT and miR-21-5p/PTEN pathways, resulting in anti-UF activity in leiomyoma cell models. Our findings suggest that THSWD could be used to treat UF.

## Introduction

Uterine fibroids (UF) are commonly occurring benign tumors of the female reproductive system [1]. They have an incidence of 70%, and 25–50% of patients exhibit clinical symptoms [2]. Anemia, menorrhagia, pain, prolonged menstruation, abnormal leucorrhea, abortion, infertility, and rectal and bladder compression symptoms are the clinical manifestations. Patients’ daily life and psychology will also be affected in severe cases [3]. The present study identified that the PI3K/Akt signaling pathway is activated in uterine fibroids [4]. PTEN, as a tumor suppressor gene, can dephosphorylate PIP3 and inhibit the activation of the PI3K/AKT pathway [5]. However, miR-21-5p can bind to and activate PTEN to release the inhibition of PTEN on the PI3K/AKT pathway [6].

In Traditional Chinese Medicine (TCM), the pathogenesis of UF may involve positive deficiency and blood stasis [7]. In the early stages of clinical practice, it has been confirmed that promoting blood circulation and removing blood stasis can control and reverse the growth of UF [8]. Taohong Siwu Decoction (THSWD) has been shown to remove blood stasis, nourish blood and promote Qi [9]. It is frequently used to treat dysmenorrhea [10] and UF [11]. Modern studies demonstrated that THSWD promotes blood circulation via a mechanism related to the PI3K/Akt signaling pathway [12].

TCM has been proven to have some advantages in the treatment of UF. Therefore, elucidating the mechanism of THSWD in UF therapy is theoretically and practically significant. This experiment will use network pharmacology to analyze the main signal pathways of THSWD intervening UF and combine existing research to screen and determine the specific pathways. It will first explain the modern mechanism of THSWD intervening UF through animal and cell experiments. To establish an experimental basis for modern TCM clinical treatment of UF.

## Materials and methods

### Screening of active compounds and targets

The TCMSP (http://tcmspw.com/) database was searched for active compounds of Taoren (*Persicae Semen*), Honghua (*Carthami Flos*), Dihuang (*Rehmanniae Radix*), Baishao (*Paeoniae Radix Alba*), Chuanxiong (*Chuanxiong Rhizoma*), and Danggui (*Angelicae Sinensis Radix*) in THSWD. Oral bioavailability (OB) ≥ 30% and drug-likeness (DL) ≥ 0.18 were the criteria for screening THSWD active compounds.

### Disease target screening

UF was searched individually in the GeneCards database (https://www.genecards.org/), OMIM database (https://omim.org/), TTD database (http://db.idrblab.net/ttd/), and DrugBank database (https://www.drugbank.ca/), and the intersection of the four databases were selected.

### Prediction of drug-disease associations

The intersection of the target of THSWD and the target of UF was obtained using the VennDiagram program package of R language.

### Construction of a component-target regulatory network

To construct the regulatory relationship network of the TCM compound, we used UniProt (https://www.uniprot.org/) data, set the species as human, searched the gene name of the target protein, and used Cytoscape 3.8.0 software.

### Construction of protein–protein interaction network

We used the STRING database (https://string-db.org) to predict the protein interaction. To build the PPI network, the UniProt accession numbers were entered into the multiple proteins search interface of the STRING database, the species was set to *Homo sapiens*, the minimum required interaction score was set to 0.9, and disconnected nodes in the network were hidden.

### GO and KEGG analysis

GO enrichment and KEGG signal pathway enrichment analyses were performed using the R language.

### Drug preparation

THSWD was composed of 9 g Taoren (*Persicae Semen*), 6 g Honghua (*Carthami Flos*), 15 g Dihuang (*Rehmanniae Radix*), 9 g Baishao (*Paeoniae Radix Alba*), 6 g Chuanxiong (*Chuanxiong Rhizoma*) and 9 g Danggui (*Angelicae Sinensis Radix*) [13]. The Chengdu University of TCM provided and authenticated the herbs. All ingredients were immersed in water for 30 min before being removed.

After boiling, the residue was filtered off and stored in a refrigerator at 4 °C for later use. Each drug was heated in warm water before use. The intragastric dose of TCM in rats was calculated as 18 g/kg, using the dose conversion coefficient of human body surface area and animal body surface area, taking 60 kg as the adult standard and the ratio of human to rat as 1:20 after conversion. The low, medium, and high doses in this study were set as 4.5, 9, and 18 g/kg, respectively.

This study used GongLiuXiao capsules (GLX capsules, Shandong Buchang Shenzhou Pharmaceutical Co., LTD.) as a positive drug. The China Food and Drug Administration (CFDA) has approved this TCM drug for treating UF. As the market has approved a drug product, the approval document is GuoYaoZhunZi-Z20055635. The drug dose of rats was calculated to be 0.9 capsule/kg after conversion using a 1:6 human-to-rat ratio.

### Construction of UF animal model

A total of 36 adult female SPF SD rats were randomly assigned to one of six experimental groups: control group (Control), model group (Model), THSWD low-dose group (THSWD-low), THSWD moderate-dose group (THSWD-moderate), THSWD high-dose group (THSWD-high), and GLX capsule group, with six rats in each group. The control group received normal saline intragastric injection treatment, while the other groups were administered intramuscular estradiol benzoate injection (0.5 mg/kg·d, Ningbo Second Hormone Factory) for 35 days. Each rat was injected with 1.0 mg progesterone every other day (Ningbo Second Hormone Factory) [14].

On the 14th day of modeling, if a disorderly arrangement of uterine smooth muscle cells, cell enlargement, and dense hypertrophy of the nucleus were found in a randomly selected rat of the model group, the modeling was considered successful. Following the establishment of the model, the rats in the control and model groups were gavaged with normal saline. The other groups continued to be injected with the model drugs. The rats were given drugs by gavage every morning for 21 days. The rats were anesthetized with sodium pentobarbital injection at the end of the treatment period. The uterus and ovaries were collected and weighed, and blood was drawn from the abdominal aorta.

### Preparation of drug-containing serum for THSWD

The serum containing THSWD and normal control serum was prepared as follows: 20 adult female SPF SD rats were randomly divided into negative serum and THSWD-serum groups, with ten rats in each group. The THSWD-serum group received 2 mL 9 g/kg d THSWD intragastric administration, while the normal control serum group received normal saline intragastric administration.

After 14 days of intragastric administration, the rats were anesthetized with pentobarbital sodium for 1 h after the last intragastric administration. The serum was separated from blood taken from the abdominal aorta. The compliment was inactivated in a 56 °C water bath for 30 min after filtration through a 0.22 μm microporous membrane to obtain THSWD-serum or normal control serum.

### Uterine and ovarian coefficient

The rats in each group were sacrificed under conventional anesthesia, the uterus and ovarian tissues were dissected, and the fat and other tissues on the tissue surface were carefully removed. The wet weight of the uterus and ovary and the uterine and ovarian coefficients were determined. The organ coefficient was calculated as the wet organ weight/body weight of rats (when sampling) × 100% [15].

### HE staining

Each rat has a section of uterine tissue from 0.5 cm above the uterine corner for this assay. The uterine tissue was immersed in the fixation solution for at least 24 h. following tissue removal, it was placed in a dehydration box and subjected to a gradient alcohol procedure. The tissue was then fixed in the wax block, which was placed on a microtome with 3 µm thickness to prepare paraffin sections. The sections were stained for 3–5 min with hematoxylin and 5 min with eosin before being sealed. Photographs were taken after HE staining.

### Electron microscopy

The tissue was treated with electron microscopy fixative before being placed at 4 °C for 2–4 h, centrifuged at 4 °C and 1500 rpm, and then rinsed thrice for 15 min each time. The solution was fixed with 1% osmic acid 0.1 M phosphate buffer, rinsed after 2 h, and then placed in 50%, 70%, 80%, 90%, 95%, and 100% alcohol, and then in 100% propanone and 100% acetone for 15 min each. This was followed by acetone permeation and an 812 embedding agent. The solution was then placed in the oven at 60 °C for 48 h. Sections with 60–80 nm thickness were made, stained, and dried overnight before microscopic examination and analysis (HT7700, Hitachi) [16].

### Immunohistochemistry

We used the instruction manual for immunohistochemical methods. The paraffin sections were dewaxed and then placed in 3% methanol hydrogen peroxide for 10 min before being washed thrice for 5 min each with Phosphate Buffer Solution (PBS). They were then immersed in 0.01 M citrate buffer and heated. After waiting for 5 min, we repeated the heating and washed the sections with PBS twice for 5 min each time.

We added blocking solution and kept them at room temperature for 20 min. The primary antibody (ab170941, 1:50, Abcam) was incubated at 4 °C overnight, and the secondary antibody (SP-9001, Beijing Zhongshan Jinqiao Biological Co., LTD) was added for incubation at 37 °C for 30 min. The color was developed at room temperature and washed after 2 min. The sections were counterstained with hematoxylin, dehydrated, rendered transparent, and sealed. A BA400Digital trimesh microcamera system (McAudi Industrial Group Co., Ltd.) was used to capture images of the sections.

### Western blot

We followed the instructions for western blotting procedures for this section.

Protein extraction. A section of uterine tissue at 0.5 cm above the uterine corner of each rat was lysed with 1 mL lysis buffer (Beyotime, Shanghai, China), centrifuged, and supernatant extracted.

Protein identification and sample denaturation. Protein concentrations were determined using the BCA protein Concentration Assay Kit (P0009, Beyotime), and the protein was denatured by heating at 95  C for 15 min.

Glue making, electrophoresis, and film turning. SDS-PAGE gel (50 μg) was used to prepare samples, which were then electrophoresed and transferred to the membrane. The electrophoresis voltage was set at 100 V for 15 min. The membrane transfer parameters were 200 mA and 1–2 h.

Blocking and antibody incubation. After 2 h of sealing with 5% TBS buffer (containing 0.05% Tween-20) and skimmed milk powder, samples were incubated with primary antibodies against PI3K (AF6241, affinity), p-PI3K (AF3241, affinity), AKT (AF6261, affinity), p-AKT (AF0016, affinity), and β-actin (AC026, abclonal). The PVDF membrane was placed on a shaker and incubated overnight at 4 °C. On day 2, the PVDF membrane was gently placed into the secondary antibody IgG (1:1000; Abcam) and incubated for 2–3 h at room temperature.

The ECL color solution was then added and allowed to react for 1 min. The exposure time was adjusted based on the signal strength.

### Quantitative real-time polymerase chain reaction (q-PCR)

We followed the general q-PCR operation instructions for this procedure.

RNA extraction. The tissue samples were homogenized after adding TRIzol reagent (vs18061730, Hefei Bomei Biotechnology Co., LTD) and chloroform. After shaking for 15 s, the samples were left for 10 min before being centrifuged for 15 min. The centrifugation conditions were 4 °C and 12,000 rpm. The samples were treated with the same amount of isopropanol (B422BA0020, Shanghai Shenggong Bioengineering Technology Service Co., LTD) for 10 min. The centrifuge was operated again at 12,000 rpm at 4 °C for 15 min. The supernatant was discarded, but the precipitated portion was retained. This was mixed with 75% ethanol before centrifuging and discarding the supernatant.

RNA reverse transcription. The Bulge-Loop^™^ miRNA RT Primer (20 µM, RiboBio) was added with RNase-free H_2_O to form a Bulge-Loop^™^ miRNA RT Primer (5 µM).

The q-PCR reaction. The experiment was carried out in a 20 µL PCR reaction system. The reaction was carried out at 95 °C for 10 min, and one cycle was performed, then 45 cycles at 95 °C, 60 °C, and 70 °C for 2 s, 20 s, and 10 s, respectively.

The U6 small nucleolar RNA gene expression was used as an internal control, and gene expression was analyzed using the 2^−ΔΔCt^ method.

The primer information is as follows:

miR-21-5p (Forward: 5ʹ—GGCGGTGTAAACATCCTT-3ʹ, Reverse: 5ʹ—GTCGTATCCAGTGCAGGGTCCGAGGTGTCG-3ʹ);

U6 (Forward: 5ʹ—CTCGCTTCGGCAGCACA-3', Reverse: 5ʹ —AACGCTTCacGAATTTGCGT-3ʹ).

### Cell culture and treatment

The optimal concentration of THSWD drug-containing serum was screened in this section. Human uterine smooth muscle cells (HUSMC, No. Cp-h053, Procell) were inoculated into 96-well culture plates at a density of 1 × 10^5^/mL to determine the optimum concentration of THSWD-serum in DMEM medium for the growth HUSMC, and then to determine the maximum non-toxic concentration of THSWD-serum. The cells were randomly divided into normal serum and THSWD-serum groups. The latter was subdivided into four concentration levels and supplemented with serum containing 20%, 15%, 10%, and 5% THSWD. The normal serum group was supplemented with 0% THSWD drug serum [17].

The optimal concentration of THSWD-containing serum was used for cell culture. We selected the 15% THSWD serum to culture tumor tissue-derived cells (No. Cp-h151, Procell) to study the effects of THSWD on cell proliferation, apoptosis, and the related mechanisms in UF. Cultured cells were then treated with miR-21-5p, NC mimic (RiboBio, Guangzhou, China), and 740Y-P (PI3K/AKT pathway activator HY-P0175, 97,709) based on the actual grouping conditions.

### CCK-8 assays

Cells in the logarithmic growth phase were washed with PBS. After digestion, the cells were centrifuged for 5 min at 1000 rpm. The supernatant was then removed, and the medium was added. Cell density was adjusted to 5 × 10^4^/mL before being added to a 96-well plate and cultured at 37 with 5% CO_2_. When the cells adhered to the wall, the corresponding drugs were divided into different groups, and the supernatant was discarded 24 h later. Each well received 110 μL of the diluted CCK-8 (70091000, Biosharp), the culture plate was gently shaken, and the culturing was continued for 2 h at 37 °C and 5% CO_2_.

A microplate reader was used to measure the absorbance value. The CCK-8 assays were used in three different experiments. CCK-8 assays were used to screen the optimal concentration of THSWD, to measure the effect of 15% THSWD-serum on the proliferation of UF cells, and to evaluate the effect of adding miR-21-5p mimics on the proliferation of UF cells.

### Flow cytometry assay

Cells in the logarithmic growth phase were washed with PBS, digested, and centrifuged for 5 min at 1000 rpm. The supernatant was discarded, and the medium was added in its place. The cell density was adjusted after counting, and the cells were cultured at 37 °C with 5% CO_2_. The cells were attached to the wall and divided into groups, and the corresponding drugs were added. After centrifugation, the supernatant was discarded to obtain a cell precipitate. Cells were resuspended with 500 µL of Binding Buffer, and then Annexin V-APC (KGA1030, keyGEN) was added, blown well, and mixed with PI. The cells were incubated at room temperature in the dark for 15 min before being stained and analyzed with a Gallios Flow Cytometer (Cytoflex, Beckman).

Cell debris and fixations were removed, and the percentage of cells in each cell cycle stage was quantified using ModFit 4.0 software. At least 10,000 cells in each sample were analyzed for a measurable signal. The apoptosis rate was calculated as the number of apoptotic cells/number of all cells × 100% [18].

### Dual-luciferase reporter assay

TargetScan databases (https://www.targetscan.org/vert_80/) predicted potential miR-21-5p binding sites to PTEN. A dual luciferase reporter assay was used to determine the potential relationship further. PTEN wild-type and mutant 3'-UTR sequences were cloned into the vector containing the luciferase reporter gene separately. Cells were collected 48 h after transfection according to the instruction for the luciferase reporter gene test kit (Promega, USA).

### Statistical analysis

All data were statistically analyzed by SPSS 21.0 and expressed as mean ± standard deviation. *P* < 0.05 values were considered statistically significant.

## Results

### Number of effective compounds in THSWD

THSWD contains 69 active compounds, including 13 from Baishao (*Paeoniae Radix Alba*), seven from Chuanxiong (*Chuanxiong Rhizoma*), two from Danggui (*Angelicae Sinensis Radix*), 22 from Honghua (*Carthami Flos*), two from Dihuang (*Rehmanniae Radix*), and 23 from Taoren (*Persicae Semen*) (Table 1).

**Table 1:** The active ingredients of THSWD

Drugs	Mol ID	Molecule Name	OB(%)	DL
Baishao	MOL001910	11alpha,12alpha-epoxy-3beta-23-dihydroxy-30-norolean-20-en-28,12beta-olide	64.77	0.38
	MOL001918	paeoniflorgenone	87.59	0.37
	MOL001919	(3S,5R,8R,9R,10S,14S)-3,17-dihydroxy-4,4,8,10,14-pentamethyl-2,3,5,6,7,9-hexahydro-1H-cyclopenta[a]phenanthrene-15,16-dione	43.56	0.53
	MOL001921	Lactiflorin	49.12	0.8
	MOL001924	Paeoniflorin	53.87	0.79
	MOL001925	Paeoniflorin_qt	68.18	0.4
	MOL001928	Albiflorin_qt	66.64	0.33
	MOL001930	Benzoyl paeoniflorin	31.27	0.75
	MOL000211	Mairin	55.38	0.78
	MOL000358	Beta-sitosterol	36.91	0.75
	MOL000359	Sitosterol	36.91	0.75
	MOL000422	Kaempferol	41.88	0.24
	MOL000492	( +)-catechin	54.83	0.24
Chuanxiong	MOL001494	Mandenol	42	0.19
	MOL002135	Myricanone	40.6	0.51
	MOL002140	Perlolyrine	65.95	0.27
	MOL002151	Senkyunone	47.66	0.24
	MOL002157	Wallichilide	42.31	0.71
	MOL000359	Sitosterol	36.91	0.75
	MOL000433	FA	68.96	0.71
Danggui	MOL000358	Beta-sitosterol	36.91	0.75
	MOL000449	Stigmasterol	43.83	0.76
Honghua	MOL001771	Poriferast-5-en-3beta-ol	36.91	0.75
	MOL002680	Flavoxanthin	60.41	0.56
	MOL002694	4-[(E)-4-(3,5-dimethoxy-4-oxo-1-cyclohexa-2,5-dienylidene)but-2-enylidene]-2,6-dimethoxycyclohexa-2,5-dien-1-one	48.47	0.36
	MOL002695	Lignan	43.32	0.65
	MOL002698	Lupeol-palmitate	33.98	0.32
	MOL002706	Phytoene	39.56	0.5
	MOL002707	Phytofluene	43.18	0.5
	MOL002710	Pyrethrin II	48.36	0.35
	MOL002712	6-Hydroxykaempferol	62.13	0.27
	MOL002714	Baicalein	33.52	0.21
	MOL002717	qt_carthamone	51.03	0.2
	MOL002719	6-Hydroxynaringenin	33.23	0.24
	MOL002721	Quercetagetin	45.01	0.31
	MOL002757	7,8-dimethyl-1H-pyrimido[5,6-g]quinoxaline-2,4-dione	45.75	0.19
	MOL002773	Beta-carotene	37.18	0.58
	MOL002776	Baicalin	40.12	0.75
	MOL000358	Beta-sitosterol	36.91	0.75
	MOL000422	Kaempferol	41.88	0.24
	MOL000449	Stigmasterol	43.83	0.76
	MOL000006	Luteolin	36.16	0.25
	MOL000953	CLR	37.87	0.68
	MOL000098	Quercetin	46.43	0.28
Dihuang	MOL000359	Sitosterol	36.91	0.75
	MOL000449	Stigmasterol	43.83	0.76
Taoren	MOL001323	Sitosterol alpha1	43.28	0.78
	MOL001328	2,3-didehydro GA70	63.29	0.5
	MOL001329	2,3-didehydro GA77	88.08	0.53
	MOL001339	GA119	76.36	0.49
	MOL001340	GA120	84.85	0.45
	MOL001342	GA121-isolactone	72.7	0.54
	MOL001343	GA122	64.79	0.5
	MOL001344	GA122-isolactone	88.11	0.54
	MOL001348	Gibberellin 17	94.64	0.49
	MOL001349	4a-formyl-7alpha-hydroxy-1-methyl-8-methylidene-4aalpha,4bbeta-gibbane-1alpha,10beta-dicarboxylic acid	88.6	0.46
	MOL001350	GA30	61.72	0.54
	MOL001351	Gibberellin A44	101.61	0.54
	MOL001352	GA54	64.21	0.53
	MOL001353	GA60	93.17	0.53
	MOL001355	GA63	65.54	0.54
	MOL001358	Gibberellin 7	73.8	0.5
	MOL001360	GA77	87.89	0.53
	MOL001361	GA87	68.85	0.57
	MOL001368	3-O-p-coumaroylquinic acid	37.63	0.29
	MOL001371	Populoside_qt	108.89	0.2
	MOL000296	Hederagenin	36.91	0.75
	MOL000358	Beta-sitosterol	36.91	0.75
	MOL000493	Campesterol	37.58	0.71

### Prediction of the key biological mechanism of THSWD responsible for controlling the development of UF

**Fig. 1:**
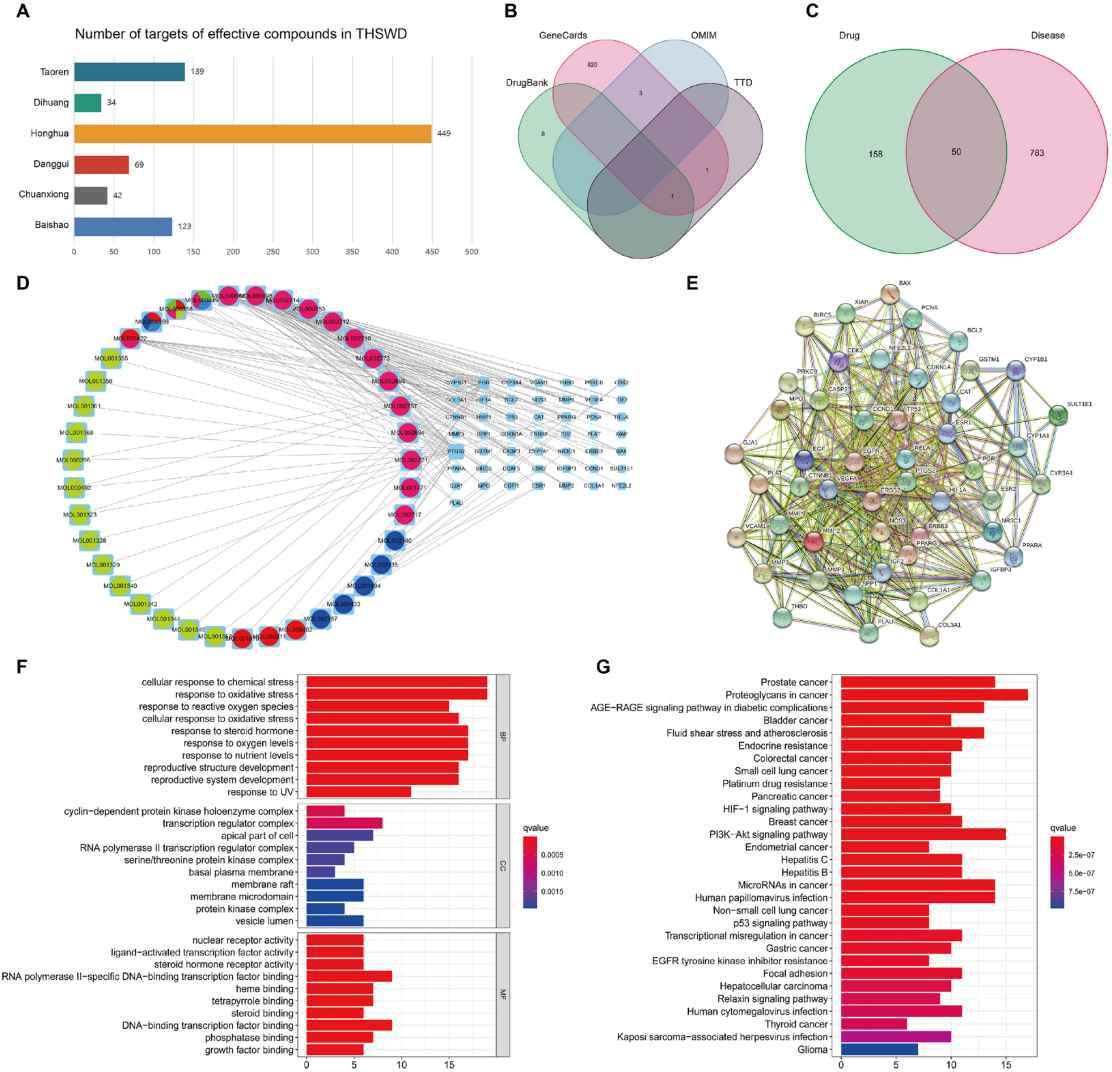
Prediction of THSWD key biological mechanisms of controlling UF development. **A** The number of targets of effective compounds in THSWD; **B** Venn diagram of UF targets; **C** Venn diagram of THSWD and UF target intersection; **D** The ingredient-target regulation network of THSWD intervention on UF; **E** PPI network graph; **F** GO enrichment analysis; **G** KEGG enrichment analysis

A total of 856 effective compound targets of six drugs were found in the TCMSP database, including 123 for Baishao (*Paeoniae Radix Alba*), 42 for Chuanxiong (*Chuanxiong Rhizoma*), 69 for Danggui (*Angelicae Sinensis Radix*), 449 for Honghua (*Carthami Flos*), 34 for Dihuang (*Rehmanniae Radix*), and 139 for Taoren (*Persicae Semen*) (Fig. 1A). There were 825 UF targets in the GeneCards database, three in the OMIM database, two in the TTD database, and ten in the DrugBank database (There are nine targets to remove the duplication); thus, 833 UF targets were obtained (Fig. 1B). After removing duplicate data from THSWD and UF, the VennDiagram program package in R language was used to find the intersection of the two. The findings revealed 50 intersecting genes (Fig. 1C).

We obtained 143 THSWD targets for treating UF, and the component-target visual regulatory network diagram was prepared using Cytoscape 3.8.0 software. Different shapes and colors represent different types of information, with blue rectangles representing genes and circles representing drug ingredients (Fig. 1D). We uploaded 50 common THSWD and UF targets into the STRING database and constructed a PPI network diagram. The network nodes represent target proteins, and the edges represent the target proteins interaction (Fig. 1E). The R language was used to analyze GO annotation information and KEGG pathway enrichment. The GO annotation obtained 1771 items, including 1586 BP, 40 CC, and 145 MF. We configured each to display the first ten items in a GO enrichment analysis diagram (Fig. 1F).

We obtained 100 results for the KEGG pathway enrichment, and the darker the color, the more significantly the gene was enriched in the pathway. The top 30 items in the KEGG enrichment analysis diagram were enriched considerably in proteoglycan, PI3K/AKT, prostate cancer, and other pathways (Fig. 1G).

### Effect of THSWD on morphological changes in rat uterine tissue

**Fig. 2:**
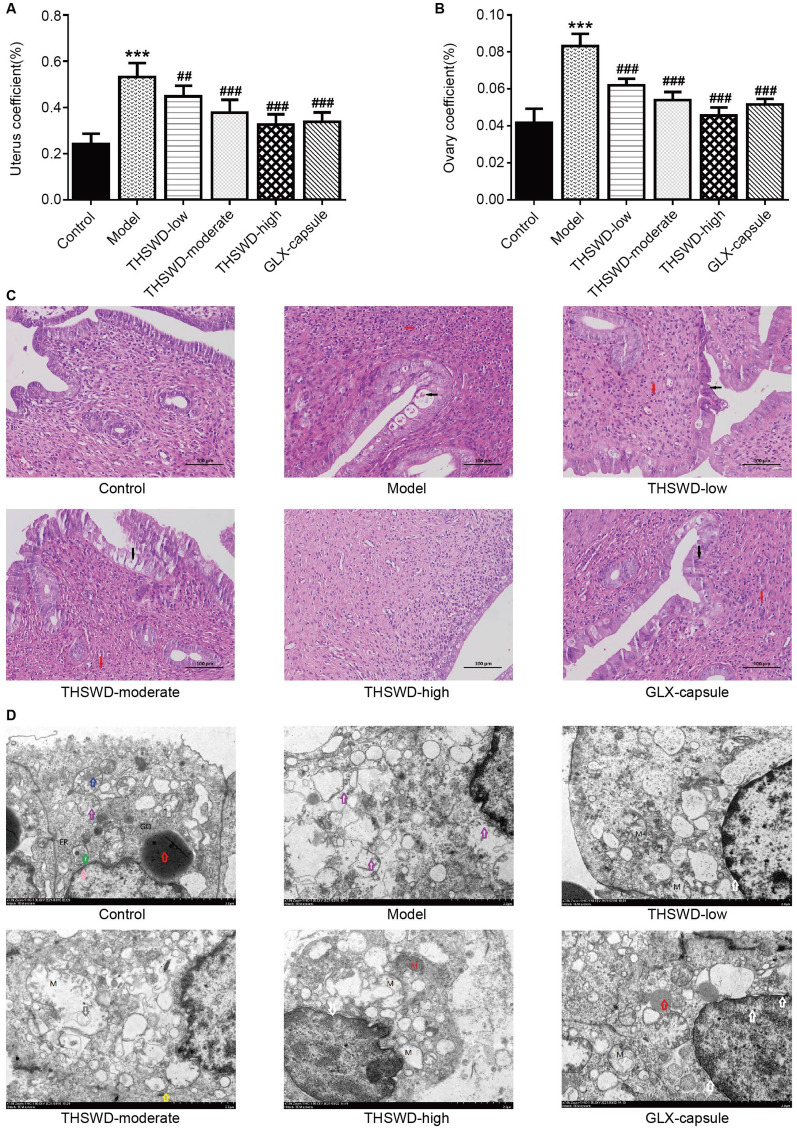
Effect of THSWD on morphological changes in the rat uterus. **A**–**B** Uterine and ovarian coefficients of rats in each group. (*n* = 6, ***p < 0.001, vs. control; ^*#*#^*p* < 0.01, ^###^*p* < 0.001, vs. model); **C** Histological changes in the rat uterus by HE staining (arrow), 200 ×, scale bar: 100 μm; **D** Uterus ultrastructure changes under an electron microscope (arrow), 7000×magnification

First, the study included uterine and ovarian coefficients. The results revealed that the uterine and ovarian coefficients in the model group were significantly higher than in the control group. Low, moderate, and high THSWD and GLX capsules doses significantly reduced the uterine and ovarian coefficients (Fig. 2A—B).

The smooth cells of the rat uteri were orderly arranged, normal in shape, and uniform in color in the control group, with no inflammatory cell infiltration or hyperplasia visible in the muscle layer. Small vesicles formed in some endometrial epithelia in the model group and eosinophilic masses were identified in the vesicles (black arrow). Neutrophils proliferated significantly in the lamina propria (red arrow). Local uterine smooth muscle hyperplasia and disorder were detected, along with increased neutrophil infiltration. THSWD-low groups were more orderly than the model group.

Some endometrial epithelia (black arrow) remained necrotic and exfoliated, neutrophils proliferated in the lamina propria (red arrow), and inflammatory cell infiltration was less intensive than in the model group. The arrangement of uterine smooth muscle cells was more orderly in the THSWD-moderate group than in the model group. Endometrial epithelial cells were occasionally necrotic (black arrow), with slight inflammatory cell infiltration (red arrow). Pathological improvement in the THSWD-high group was comparable to that in the control group, and no obvious inflammatory cell infiltration was observed. The GLX-capsule group had less neutrophil infiltration (black arrow) than the model group, there were neutrophils in the lamina propria (red arrow), and the smooth muscle of the myometrium was regularly arranged (Fig. 2C).

The ultrastructure of uterine tissue in each group was then examined using an electron microscope. The findings revealed that the cellular connections between the endometrial epithelial cells of the control group were clear, and the mitochondrial structure in the cytoplasm was normal. In the model group, the secretory nucleus volume of endometrial epithelium decreased. Moreover, many secretory granules with high electron density were observed in the cytoplasm. Lipid droplets were seen in the cells, and the mitochondrial crest shrank or disappeared, resulting in low electron density swelling. The THSWD-low group had slightly lower cell nuclei of endometrial epithelium than the model group. Low electron density swelling was observed in the mitochondrial crest structure.

There were secretory granules with medium electron density in the cytoplasm, and the connections between the cells were still somewhat blurred. The cytoplasmic content of epithelial cells decreased in the endometrial epithelium of the THSWD-moderate group. The nuclei were densely packed, and numerous intracellular mitochondria were swollen. The crista fracture disappeared as a low electron density vacuole, and intercellular connections were visible. The nuclei of endometrial epithelium formed multiple depressions in the THSWD-high group.

The nuclear envelope was dilated locally, the perinuclear space was widened, and mitochondria with the normal structure were identified. Some mitochondria were swollen with low electron density. The endometrium epithelial nuclei were normal in the GLX-capsule group, but the nuclear membrane was still dilated, and the gap was locally widened. Some mitochondrial structures were normal. Simultaneously, many mitochondrial cristae fractures vanished, revealing low electron density swelling, and secretory granules with high electron density were observed in the cytoplasm (Fig. 2D).

### Effect of THSWD on the PI3K/AKT pathway and miR-21-5p/PTEN axis in rat uterine tissue

**Fig. 3:**
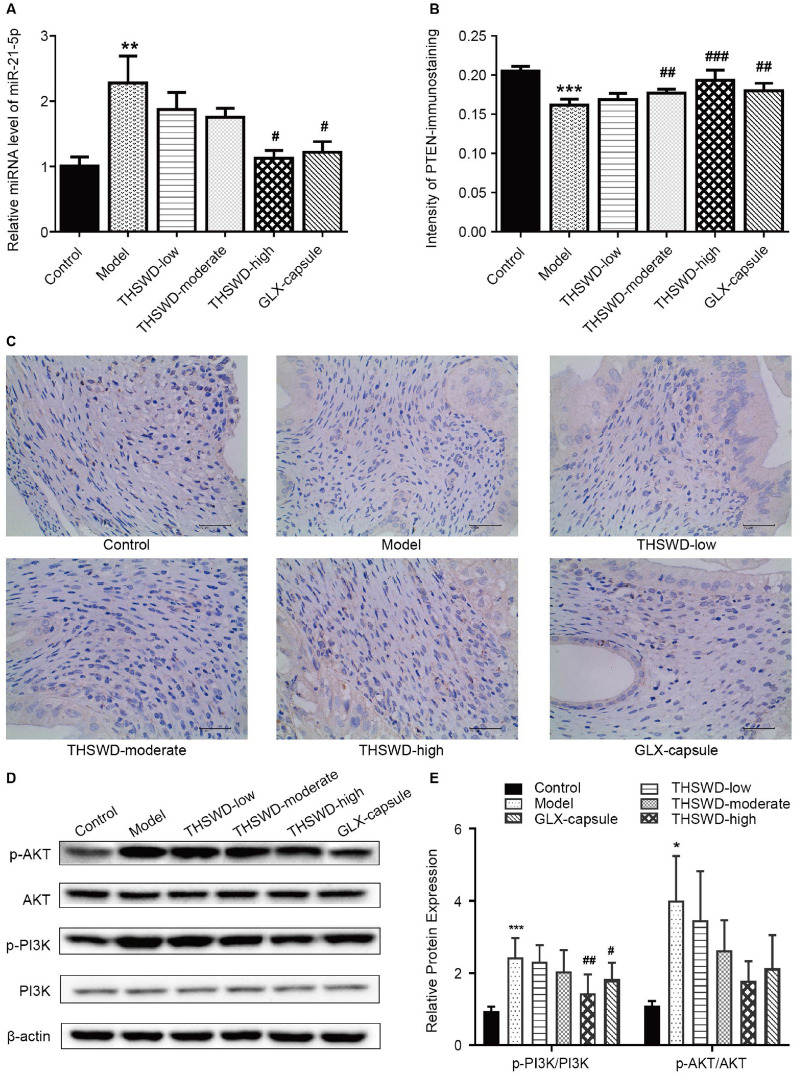
Effect of THSWD on the PI3K/AKT pathway and the miR-21-5p/PTEN axis in rat uterine tissue. **A** The expression level of miR-21-5p in uterine tissues by q-PCR; **B**–**C** Immunohistochemical staining of PTEN in uterine tissues by (40 ×); **D**–**E** Expression levels of p-PI3K/PI3K and p-AKT/AKT by western blot. (*n* = 6, *p < 0.05, **p < 0.01, ***p < 0.001, vs. control; ^#^*p* < 0.05, ^##^*p* < 0.01, ^###^*p* < 0.001, vs. model)

Q-PCR was used to detect the expression level of miR-21-5p in the uterine tissues of each group. It was found to be significantly higher in the model group compared to the control group. Compared with the model group, miR-21-5p expression levels in the THSWD-high and GLX capsule groups were significantly lower (Fig. 3A).

The immunohistochemical staining revealed that PTEN expression was significantly lower in the model group than in the control group. The PTEN expression was significantly higher in the THSWD-moderate/high group and the GLX capsule group compared to the model group (Fig. 3B–C).

The western blot results revealed that the expression levels of p-PI3K/PI3K and p-AKT/AKT were significantly higher in the model group compared to the control group. The high THSWD and GLX capsule doses significantly reduced p-PI3K/PI3K expression levels compared to the model group. Despite the lack of a statistical difference, the expression of p-AKT/AKT in the THSWD-low/moderated/high group and the GLX capsule group declined (Fig. 3D–E).

### Effects of THSWD medicated serum on the proliferation and apoptosis of human UF tissue-derived cells by regulating the PI3K/AKT pathway

**Fig. 4:**
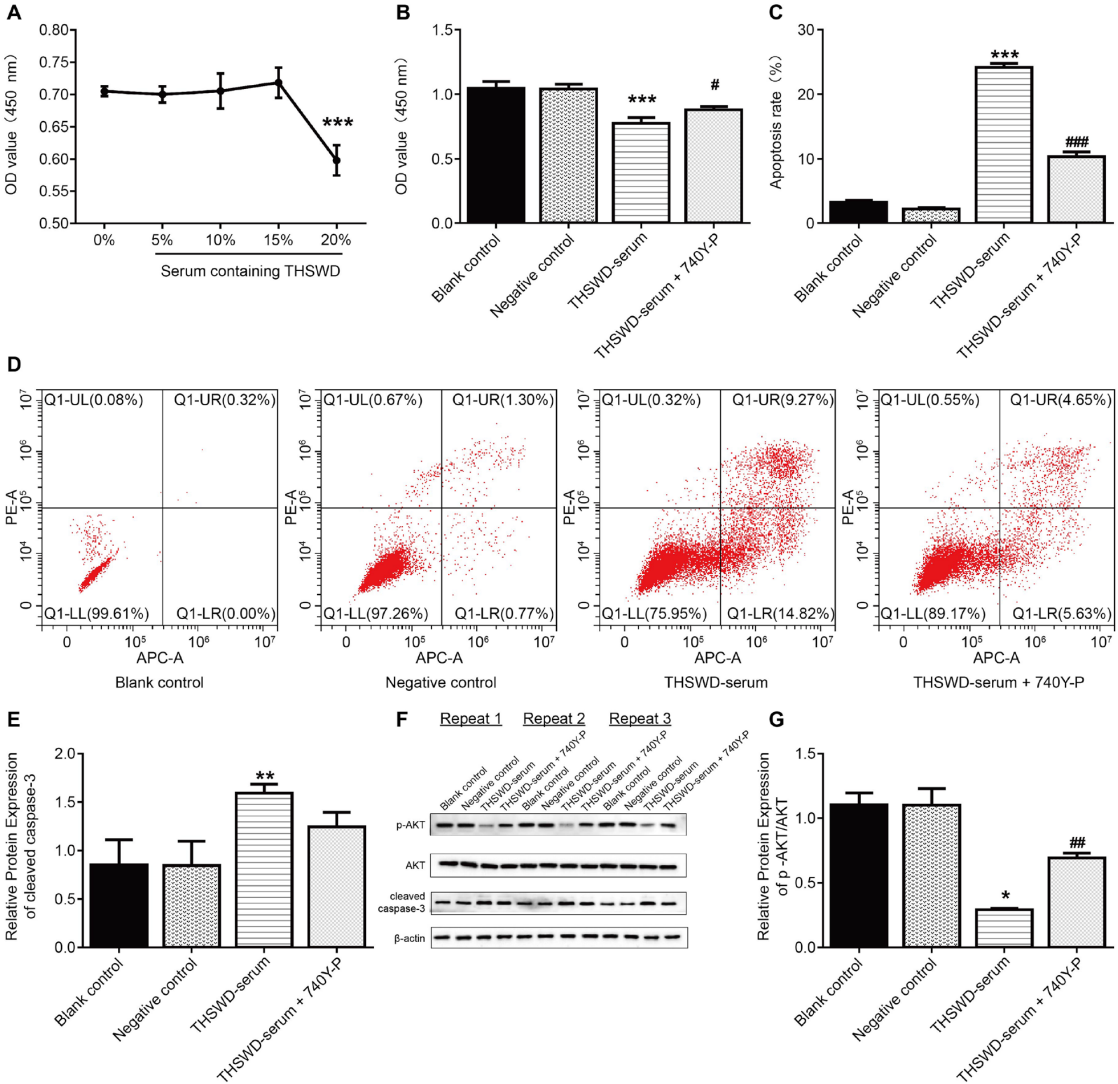
THSWD medicated serum influences the proliferation and apoptosis of human UF tissue-derived cells via the PI3K/AKT pathway. **A** The effects of different drug serum concentrations on cytotoxicity by CCK-8 assay. ***p < 0.001, vs. 0% THSWD-serum; **B** Proliferative activity of tumor cells by CCK-8 assay; **C**–**D** Apoptosis rate of cells derived from human UF tissue by flow cytometry; **E**–**G** Expression levels of cleaved caspase-3 and p-AKT/AKT by western blot (*n* = 3, *p < 0.05, **p < 0.01, ***p < 0.001, vs. negative control; ^#^*p* < 0.05, ^##^*p* < 0.01, ^###^*p* < 0.001, vs. THSWD-serum)

CCK-8 assay was used to detect HUSMC activity and determine the maximum non-toxic concentration of THSWD-serum. The results revealed that 20% THSWD-serum significantly reduced the HUSMC cell proliferation (Fig. 4A). Therefore, we selected 15% THSWD-serum to investigate the effect and mechanism of THSWD on the development and growth of cells derived from human UF. The CCK-8 assay was also used to assess the proliferation. The CCK-8 assay was used again to assess the effect of 15% THSWD-serum on UF cell proliferation. Cell proliferation was significantly reduced in the THSWD-serum group compared to the negative control group (Fig. 4B).

Concurrently, flow cytometry results revealed that THSWD-serum significantly increased cell apoptosis (Fig. 4C–D). However, the 740Y-P treatment reversed all these values (Fig. 4B–D).

The western blot results indicated that cleaved caspase-3 expression levels were significantly increased after THSWD-serum treatment, but the opposite trend was observed after the 740Y-P treatment. The expression level of p-AKT/AKT decreased significantly after treatment with THSWD-serum, which was reversed by further treatment with 740Y-P (Fig. 4E–G).

### Effects of THSWD-serum mediated by the miR-21-5p/PTEN axis on the proliferation and apoptosis of human UF tissue-derived cells

**Fig. 5:**
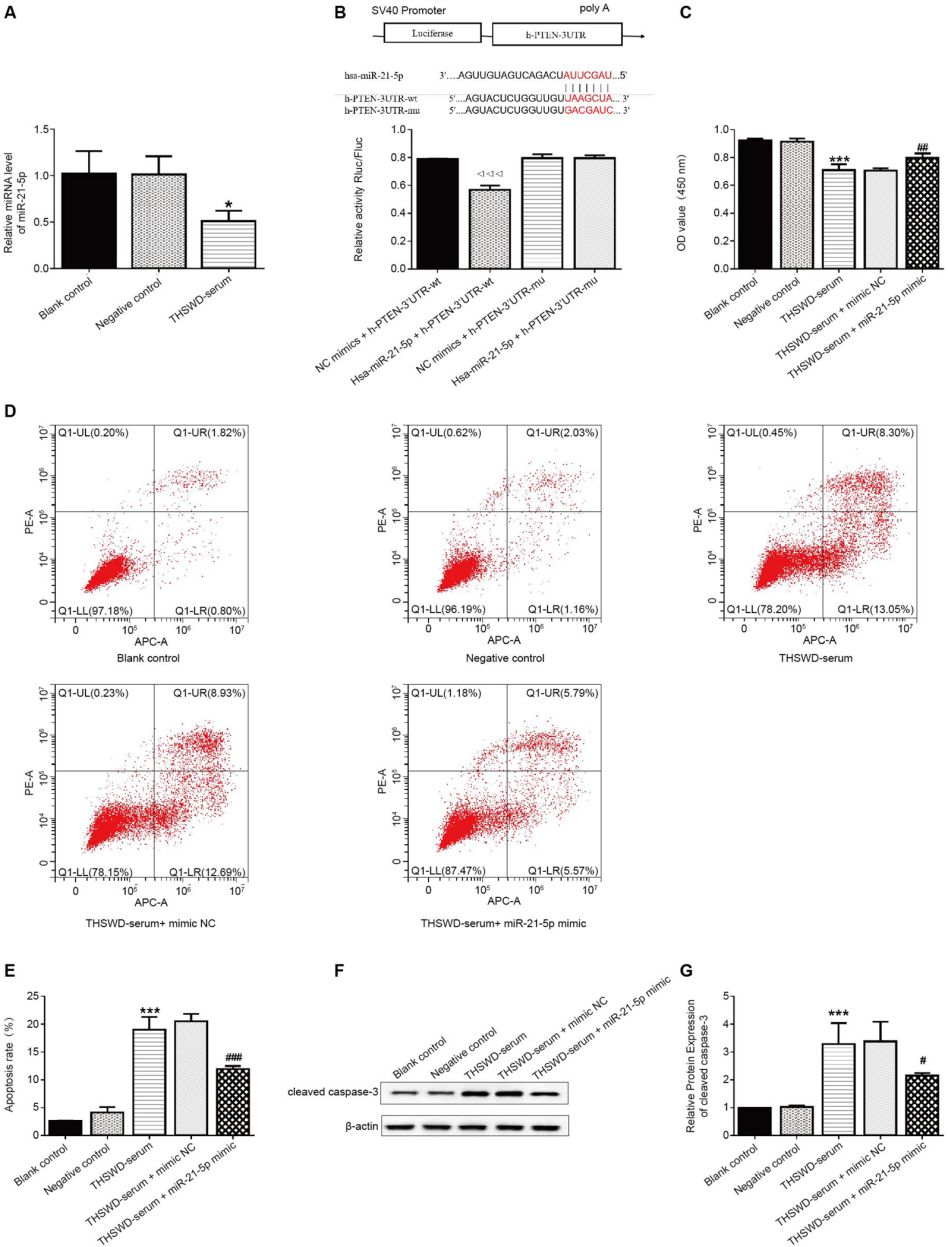
THSWD-serum effects on proliferation and apoptosis of human UF tissue-derived cells via the miR-21-5p/PTEN axis. **A** Expression of miR-21-5p in human UF tissue-derived cells by q-PCR; **B** The interaction between miR-21-5p and PTEN was confirmed by luciferase reporter assay; **C** Proliferative activity of cells derived from human UF tissues by CCK-8 assay; **D**–**E** Apoptosis rate of cells derived from human UF tissues by flow cytometry; **F**–**G** Expression levels of cleaved caspase-3 by western blot. (*n* = 3, *p < 0.05, ***p < 0.001, vs. negative control; ^△△△^*p* < 0.001, vs. NC mimics + h-PTEN-3’UTR-wt; ^#^*p* < 0.05, ^##^*p* < 0.01, ^###^*p* < 0.001, vs. THSWD-serum)

The relative expression of miR-21-5p in cells derived from human UF was assessed using q-PCR. The expression levels of miR-21-5p were significantly lower in the THSWD-serum group than in the negative control group (Fig. 5A). The results of dual-luciferase reporter assay demonstrated that miR-21-5p mimics significantly reduced the luciferase activity of PTEN-wt (Fig. 5B). The CCK-8 results further revealed that cell proliferation was significantly reduced in the presence of THSWD-serum compared to the negative control group. However, adding the miR-21-5p mimics partially reversed this decline (Fig. 5C). Flow cytometry results revealed that THSWD-serum treatment significantly increased cell apoptosis. In contrast, the processing of miR-21-5p mimics reversed the promotor effect of THSWD-serum on cell apoptosis (Fig. 5D–E).

Western blot was used to examine the protein expression level of cleaved caspase-3. THSWD-serum treatment significantly increased cleaved caspase-3 expression (P < 0.01) when compared to the negative control group, which was partially reversed by the miR-21-5p mimic (Fig. 5F–G).

### THSWD-serum regulated the PI3K/AKT pathway mediated by the miR-21-5p/PTEN axis in human UF tissue-derived cells

**Fig. 6:**
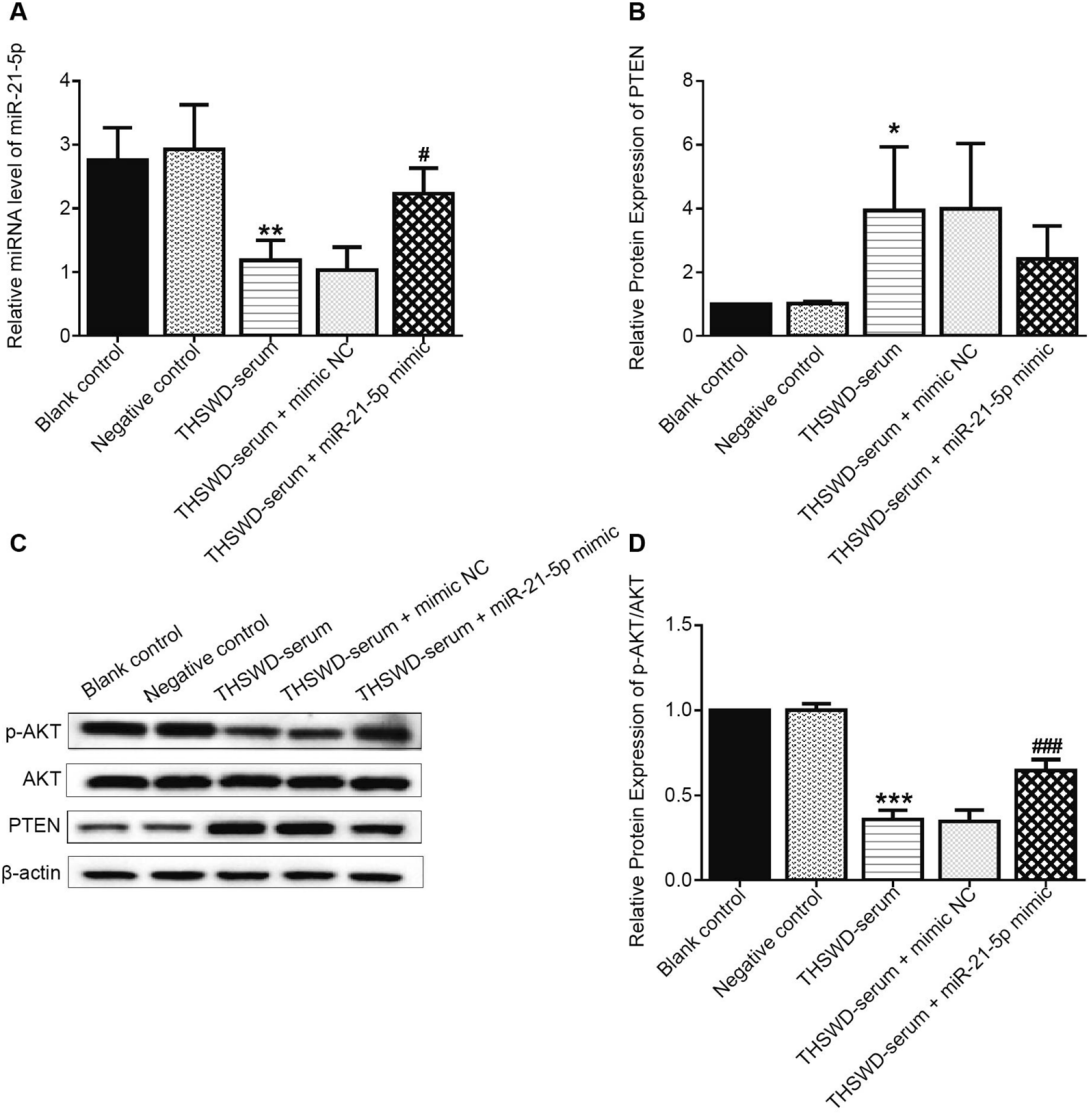
THSWD-serum regulated the PI3K/AKT pathway in human UF tissue-derived cells via the miR-21-5p/PTEN axis. **A** q-PCR analysis of miR-21-5p expression in human UF tissue-derived cells; **B**–**D** Expression levels of PTEN and p-AKT/AKT by western blot (*n* = 3, *p < 0.05, **p < 0.01, ***p < 0.001, vs. negative control; ^#^*p* < 0.05, ^###^*p* < 0.001, vs. THSWD-serum)

The q-PCR results revealed that the expression of miR-21-5p was significantly lower in the THSWD-serum group compared to the negative control group but significantly higher after the subsequent miR-21-5p transfection (Fig. 6A).

The western blot analysis revealed that PTEN expression was significantly increased after THSWD-serum treatment, and transfection of miR-21-5p mimic could reduce the above result. However, THSWD-serum treatment resulted in significantly lower expression of p-AKT/AKT. Meanwhile, miR-21-5p mimic transfection partially eliminated the effect (Fig. 6B–D).

## Discussion

The incidence of UF has been steadily increasing in recent years. This condition is rare before puberty and progresses to atrophy or regression after menopause. It is more in women of childbearing age and is characterized by rapid proliferation of formed UF [19]. Drug therapy and surgery are the most common forms of treatment; however, their side effects are severe [20]. Therefore, finding safe and effective methods to preserve the uterus to the greatest extent possible has become the primary research focus in recent years. The effect of THSWD on UF and its mechanism were investigated in this study to lay the theoretical foundation for its clinical application.

In China, increasing blood circulation and removing blood stasis have been proven effective in treating UF. Qing Dynasty [21], THSWD was first reported in Wuqian's book Yizong Jinjian, a classic recipe for activating blood and removing blood stasis. Modern research reveals that it can promote blood circulation and improve diseases caused by blood stasis, which is highly consistent with the etiology and pathogenesis of UF, according to TCM [22, 23]. Therefore, THSWD is frequently used in clinical medicine in China to treat UF [24, 25]. In addition, THSWD has been found to have antitumor activity in other cancers. Early studies demonstrated the anti-B16 melanoma effect of THSWD [26]. THSWD also had an excellent therapeutic effect on breast cancer [27], while other studies reported that THSWD-serum inhibited breast cancer cell proliferation [28].

THSWD contained 69 active compounds, according to the results of the network pharmacological analysis. There were 50 overlapping genes between the peach and UF targets. PPI network analysis revealed higher TP53, VEGFA and ESR1 count values. The GO annotation obtained 1771 entries, 1586 of which were BP, 40 CC, and 145 MF. The KEGG pathway enrichment yielded 100 results, and the darker the color, the more significant the gene enrichment in the pathway. The findings revealed that genes in PI3K/Akt, proteoglucan, AGE/RAGE, and other pathways were significantly enriched. Previous studies have shown that the PI3K signaling pathway is overexpressed in UF [29]. We chose the PI3K/Akt signaling pathway for the experimental research based on the results of this network pharmacological analysis.

HE staining and electron microscope examination in animal experiments revealed that this prescription could effectively improve the pathological morphology of the uterus. The expressions of miR-21-5p, P-PI3K/PI3K, and P-Akt/Akt were decreased in the administration group, while PTEN expression was increased. The results of the cell experiments indicate that THSWD-serum treatment significantly reduced tumor cell survival and increased cleaved caspase-3 protein expression, implying that THSWD can inhibit tumor cell proliferation.

P13K as a second intracellular messenger, further activated downstream AKT and stimulated its regulation of apoptosis factor-cleaved caspase-3 in the PI3K/AKT signaling pathway [30]. PTEN is a bispecific phosphatase that removes phosphate from PI3K, inhibiting the activation of the PI3K/AKT signaling pathway [31] and acting as a tumor suppressor [32]. Targetscan, an online binding site prediction database, and previous studies have revealed that miR-21-5p directly targets and binds to PTEN [33]. In addition, multiple studies have highlighted that miR-21-5p is up-regulated in UF [34, 35], which can promote fibroid cell proliferation [36]. Our findings support this. We found that miR-21-5p has a targeting relationship with PTEN in UF and can regulate the PI3K/Akt signaling pathway through experimental detection. THSWD has intervened in this process.

THSWD inhibited UF proliferation and induced tumor cell apoptosis via the PI3K/AKT pathway mediated by the miR-21-5p/PTEN axis. However, there have been few modern pharmacological studies on THSWD, and only a few experiments have independently confirmed its treatment effect for UF. Therefore, the mechanism of action of the main components of THSWD in treating UF requires further investigation.

## Conclusions

**Fig. 7:**
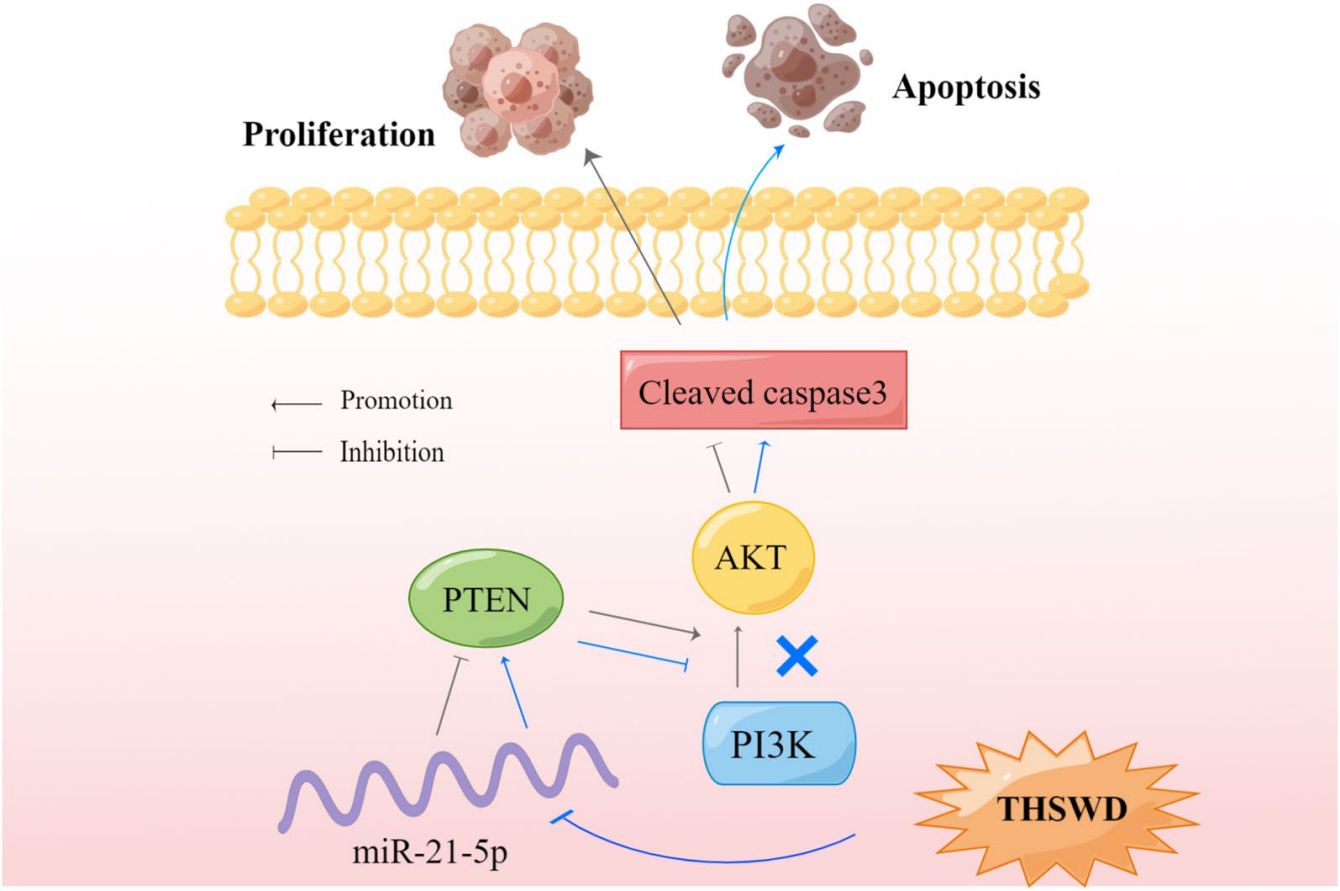
Mechanism of THSWD in the treatment of UF

This study used network pharmacology to demonstrate that PI3K/AKT is a potential THSWD pathway for treating UF. In vivo and in *vitro* experiments, we confirmed that PI3K/AKT is highly expressed in UF. THSWD inhibits the activation of the PI3K/AKT pathway through the miR-21-5p/PTEN axis, weakening tumor cell proliferative ability and inducing apoptosis (Fig. 7), which is the key mechanism of THSWD in the treatment of UF.

This study inevitably has some limitations. We demonstrated that THSWD could effectively intervene in UF via the PI3K/AKT pathway, revealing the primary pathogenesis of UF and the intervention pathway of its treatment drugs. Because different Chinese herbs are combined in clinical practice, the effective chemical components of THSWD were not analyzed in our experiment. In the near future, we aim to investigate further which components of THSWD are important in treating UF.

## Data Availability

The datasets used during the present study are available from the corresponding author on reasonable request.

## Abbreviations

UF: Uterine fibroids
THSWD: Taohong Siwu Decoction
TCM: Traditional Chinese medicine
OB: Oral bioavailability
DL: Drug-likeness
GLX: GongLiuXiao capsules
CFDA: China Food and Drug Administration
PBS: Phosphate buffer solution
Q-PCR: Quantitative real-time polymerase chain reaction
HUSMC: Human uterine smooth muscle cells
